# Socioeconomic Determinants of Participation in Cancer Screening in Argentina: A Cross-Sectional Study

**DOI:** 10.3389/fpubh.2021.699108

**Published:** 2021-08-24

**Authors:** Bernardo Nuche-Berenguer, Dikaios Sakellariou

**Affiliations:** ^1^London School of Hygiene and Tropical Medicine, London, United Kingdom; ^2^School of Healthcare Sciences, Cardiff University, Cardiff, United Kingdom

**Keywords:** breast cancer, cancer screening (MeSH), cancer disparities, cervical cancer, colorectal cancer

## Abstract

Low socioeconomic status is associated with late cancer diagnosis and mortality in Argentina. It is important that cancer screening services are accessible to the whole population so that cancer can be detected early. Our aim in this study was to investigate socioeconomic determinants for the disparities in the use of breast, cervical, and colorectal cancer screening services in Argentina, and to measure the country progress in reducing differences in cancer screening participation across socioeconomic levels. We performed a secondary analysis of cross-sectional data from the 2018 National Survey of Risk Factors of Argentina. The sample included data from 49,170 households. We also compared the results with data from the 2013 wave of the same survey in order to assess progress on cancer screening participation across income and education categories. Income, education, health insurance, disability, and marital status were associated with cancer screening underuse in Argentina. Comparison between 2013 and 2018 demonstrated that there has been some progress toward increasing cancer screening uptake, but this increase is not equitably distributed across the population. To further reduce disparities in cancer participation across socioeconomic levels, cancer screening programs in Argentina should reinforce strategies to become more accessible. It is important to proactively reach those populations that are underusers of cancer screening and ensure that barriers that stop people from accessing cancer screening are explored and adequately addressed.

## Introduction

There is a growing body of evidence on the relationship between socioeconomic status and cancer disparities, mostly from high-income countries (1). While the early detection of some cancer types such as breast (2, 3), cervical (4), and colorectal (5, 6) cancer is reported to have a positive impact on mortality reduction, access to screening services is not always equitably distributed. Low income, low education level, lack of health insurance, and single marital status are associated with underuse of cancer screening services in Latin America (7).

Argentina is an upper middle-income country (www.datahelpdesk.worldbank.org) that has universal health coverage. However, the characteristics of health services in terms of quality and equity are variable (8), partly due to the fragmentation of the health system (9). It is estimated that there are 130,000 new cases and 69,000 deaths from cancer each year in Argentina, while projections indicate a 24% increase in cancer incidence and a 26% increase in cancer mortality by 2030 (10). Late diagnosis and mortality from cancer in the country are more common in lower socioeconomic strata (11–14). In line with international recommendations, Argentina established national screening programs for cervical cancer in 1998 (and reformed in 2008), for breast cancer in 2011, and for colorectal cancer in 2013 (15). For these programmes to be effective in reducing cancer morbidity and mortality, a significant proportion of the population needs to participate (16). Previous analyses reported differences in the participation in these programmes across socioeconomic levels (17–20). However, these analyses have three main limitations: first, they were conducted with data from before 2009 and may not reflect the impact that new cancer control initiatives, such as the establishment or reforms of national cancer screening programs (21–23), the progressive introduction of molecular Human Papillomavirus (HPV) testing (24), and the creation of the National Cancer Institute in 2010. Second, they did not disaggregate data by disability, despite known associations between disability and cancer screening both in Latin America and globally (25–27). Third, there are no available studies on the utilization of colorectal cancer screening across socioeconomic levels in Latin America (7). This is particularly relevant for Argentina, where colorectal cancer ranks second among the most common cancer types (10).

The aim of this study was to investigate the socioeconomic determinants underlying disparities in the utilization of cancer screening services in Argentina and to measure the country progress in reducing such disparities across socioeconomic levels. The specific objectives were to:

1) Measure breast, cervical, and colorectal cancer screening underuse across different socioeconomic strata and for people with disabilities using data from 2018.
2) Compare self-reported participation on cancer screening from 2013 and 2018 to evaluate progress toward widening screening uptake, given that many programmatic changes in cancer prevention have been introduced since 2013.

## Methods

### Study Design

We performed a secondary analysis of cross-sectional data from the 2018 National Survey of Risk Factors of Argentina (known as ENFR in Spanish) (28), and we also compared this with data from the same survey that was conducted in 2013 in a different sample. All microdata obtained from the ENFR were freely available in the public domain (29). The Research Governance & Integrity Office of the London School of Hygiene and Tropical Medicine assessed that this project did not require ethical approval.

The ENFR is a cross-sectional, household interview survey, which is conducted by the National Ministry of Health every five years (first in 2005 and then in 2009, 2013, and 2018) and is representative of the Argentinean urban population aged 18 and over, living in towns/cities with 5,000 or more inhabitants. Data for the 2018 ENFR were collected between September and December 2018. The survey uses a probabilistic, stratified, and multistage sampling design. The sampling is done from an Urban Sampling Framework built by the National Institute of Statistics and Censuses. The 2018 survey applied STEPS, the World Health Organization approach to chronic disease risk factors surveillance (30). The first step consisted of a questionnaire administered by an interviewer, covering aspects related to socioeconomic conditions as well as health behaviors. The second and third steps consisted of a number of biometrical and biochemical measures respectively. A detailed description of the survey methodology can be found elsewhere (28, 31). In this study, we only used information obtained during step 1.

The sample obtained for this step in the 2018 survey was from 49,170 households at the national level and the response rate was 73.4% (31). Each head-of-household was interviewed to collect information about the dwelling, and one individual over 18 was randomly selected applying a Kish selection table to answer the questionnaire (32). The questions about breast and cervical cancer screening were answered exclusively by women of every age, the questions on colorectal cancer screening participation in the last two years were answered by both men and women of every age, and the question about ever participation in colorectal cancer screening were answered exclusively by men and women over 50. For the purpose of the study 7,070 women older than 50 responded the questions related to the use of mammography; 11,602 women over 35 years old responded the questions on cervical cancer screening and 12,122 persons (both men and women) responded the questions on colorectal cancer screening.

### Dependent Variables

The ENFR questionnaire covers basic sociodemographic and health status information, and specific questions on health-risk factors (28), including the following questions on cancer screening:

- Use of mammography: “Have you ever had a mammography?” and “Did you have a mammography in the last two years?”
- Use of Pap smear: “Have you ever had a Pap smear?” and “Did you have a Pap smear in the last two years?
- Use of colorectal cancer screening: Have you ever been screened for colorectal cancer?”; “Have you ever been examined to detect polyps or colorectal cancer with a fecal occult blood test?”; “Have you ever been examined to detect polyps or colorectal cancer with a colonoscopy?”; Have you ever been examined to detect polyps or colorectal cancer with a barium enema?” “When was the last time you were screened for colorectal cancer?”

Based on the participants' answers to the questions above, we built six main dependent variables of cancer screening underuse that were as follows:

- For breast cancer screening: no mammography in the previous 2 years and no mammography ever (women over 50).
- For cervical cancer screening: no Pap smear in the previous 2 years and no Pap smear ever (women over 35).
- For colorectal cancer screening: no colorectal cancer screening in the previous 2 years (by any of the three available methods) and no colorectal cancer screening ever (men and women over 50).

The decisions on the screening intervals that were considered to define cancer screening underuse, as well as the age limits used, were based on the Argentinian guidelines (15). These guidelines recommend initiating breast and colorectal cancer screening at age 50, and cervical cancer screening at age 35, and were consistent with those used in other countries in Latin America (7).

### Independent Variables

We used the sociodemographic variables associated with cancer screening participation in Latin America (7). We included household income (categorized as quintiles and adjusted per consumption unit), education level, health insurance status, physical disability, region of residence (by the six regions in the country), province of residence (24 provinces that are within the six regions), the size of the city of residence, self-report health status, age and marital status. The education level variable had three categories: low level (primary education not completed), medium level (primary education completed but incomplete secondary education) and high level (completed secondary education and beyond). The marital status variable was generated from information in the questionnaire and divided into two categories: single (including divorced people) and not single (either married or with a stable partner). To measure physical disability, we used as a proxy “capacity to walk” which had as available answers full, medium, and null. Full ability to walk was categorized as no disability (none), medium as moderate, and null ability to walk was categorized as severe disability. For the analysis of the colorectal cancer screening, gender (men/women), was also included as an independent variable. All variables were categorized and treated as dummy variables.

### Data Analysis

We used Stata 14.2 to conduct logistic regressions to assess the impact of socioeconomic determinants on the underuse of breast, cervical, and colorectal cancer screening. First, the percentage of self-reported non-participation in cancer screening was estimated for the two measures of underuse for breast, cervical, and colorectal cancer and measured for each of the independent variables described above. Secondly, we used multiple logistic regressions adjusted for income, age, region of residence, education level, health insurance status, physical disability, marital status, and self-reported health status, and we calculated the adjusted odds ratios (OR) as well as 95% confidence intervals. For the analysis, we applied the survey expansion factor described in the survey methods (33). The survey expansion factor is based on the idea that each selected household represents a number of households of similar characteristics. It allows the extrapolation of the survey results by applying the inverse probabilities of inclusion of each household in the survey.

In order to assess progress toward widening screening uptake, we compared the results with those obtained by the 2013 ENFR, which followed a similar methodology and was conducted in 46,555 households with a response rate of 70.7% (33). Some of the variables of the 2013 ENFR survey had to be recoded in order to be comparable with those of the 2018 ENFR survey. We compared the self-reported participation on cancer screening and performed a chi-squared test to assess statistical significance. Moreover, to assess progress on cancer screening participation across socioeconomic levels, we compared the OR of the two measures of breast, cervical, and colorectal cancer screening underuse across the 2013 and 2018 ENFR surveys.

## Results

### Socioeconomic Determinants for Underuse of Cancer Screening in Argentina

Table 1 shows the descriptive socioeconomic characteristics for breast, cervical, and colorectal cancer screening underuse among target populations. The full sample characteristics can be seen in Supplementary Materials 1–3.

**Table 1:** Descriptive socioeconomic characteristics for breast, cervical, and colorectal cancer screening underuse in Argentina.

	**Mammography** **(women over 50**, ***n*****= 7,070)**		**Pap smear** **(women over 35**, ***n*****= 11,602)**		**Colorectal cancer screening** **(men/women over 50**, ***n*****= 12,122)**	
	**Never screened (%)** **(*n* = 1,321)**	**Not screened in past 2 years (%)** **(*n* = 3,064)**	**Never screened (%)** **(*n* = 1,323)**	**Not screened in past 2 years (%)** **(*n* = 4,587)**	**Never screened (%)** **(*n* = 8,405)**	**Not screened in past 2 years (%)** **(*n* = 10,055)**
**Income (Quintiles)**						
1st	31.88	54.55	18.11	47.56	79.75	89.28
2nd	24.58	51.69	14.85	46.93	74.90	86.99
3rd	18.25	44.14	10.54	40.19	71.02	83.68
4th	13.71	40.27	8.22	36.50	66.43	80.51
5th	8.40	28.16	6.09	27.21	59.74	76.76
**Education level**						
Low	35.82	64.0	24.68	61.62	75.20	86.79
Medium	20.99	48.53	13.06	47.59	72.25	85.11
High	10.07	30.78	7.29	29.19	64.91	79.43
**Health insurance**						
Yes	16.85	41.53	10.17	38.15	67.02	81.48
No	30.60	55.11	16.08	44.80	83.47	90.88
**Marital status**						
Not single	14.73	34.55	8.79	32.25	68.13	80.79
Single	21.17	48.85	15.71	45.96	71.07	85.03
**Physical disability**						
None	10.54	35.70	11.89	42.40	71.07	83.01
Moderate	14.40	53.80	14.60	56.80	67.69	82.95
Severe	24.49	76.53	24.44	78.88	69.01	83.21
**Gender**						
Women					70.07	84.59
Men					69.07	80.77

*The income quintiles were (in Argentinian pesos): 1st quintile (0–8,599); 2nd quintile (8,600–14,999); 3rd quintile (15,000–19,999); 4th quintile (20,000–29,999); 5th quintile (30,000 and above)*.

The highest rates of underuse were observed for colorectal cancer screening and the lowest for cervical cancer. The results showed a lower percentage in the underuse of all types of cancer screening in the wealthiest and most educated population. These lower underuse rates were to a lesser extent also observed among people with health insurance. For breast and cervical cancer screening, being single was apparently related to lower use of screening services. People with disabilities had lower participation on breast and cervical cancer screening, and this was associated with the degree of disability.

### Measure of Mammography Underuse

Seven thousand and seventy women older than 50 responded the questions related to the use of mammography. As shown in Table 2, lower underuse of mammography was observed in women with a higher income and education level. Highest-income women (quintile 5) were less likely to have never undergone mammography in the past or in the previous 2 years. Education level had a similar effect, with women with high education reporting lower rates of underuse. Women who had no health insurance had higher odds to never having undergone a mammography and not having undergone a mammography in the previous 2 years. Finally, women with severe physical disability had higher odds to having never undergone a mammography and or not having undergone one in the previous 2 years.

**Table 2:** Socioeconomic characteristics associated with mammography, Pap smear, and colorectal cancer screening underuse.

**Mammography (** ***n*** **= 7,070)**					**Pap Smear (** ***n*** **= 11, 602)**				**Colorectal cancer screening (** ***n*** **= 12,122)**			
	**Never screened** **(** ***n*** **= 1,321)**		**Not screened in past 2 years** **(** ***n*** **= 3,064)**		**Never screened** **(** ***n*** **= 1,323)**		**Not screened in past 2 years** **(** ***n*** **= 4,587)**		**Never screened** **(** ***n*** **= 8,405)**		**Not screened in past 2 years** **(** ***n*** **= 10,055)**	
	**OR**	**95% CI**	**OR**	**95% CI**	**OR**	**95% CI**	**OR**	**95% CI**	**OR**	**95% CI**	**OR**	**95% CI**
**Income (Quintiles)**												
1st	1.000	1.000	1.000	1.000	1.000	1.000	1.000	1.000	1.000	1.000	1.000	1.000
2nd	0.912^*^	0.905–0.918	0.986^*^	0.980–0.992	0.732^*^	0.728–0.737	0.932^*^	0.928–0.937	0.786^*^	0.782–0.791	0.973^*^	0.967–0.980
3rd	0.611^*^	0.606–0.616	0.761^*^	0.756–0.766	0.431^*^	0.427–0.433	0.653^*^	0.650–0.656	0.694^*^	0.690–0.697	0.787^*^	0.782–0.793
4th	0.553^*^	0.548–0.558	0.723^*^	0.718–0.728	0.429^*^	0.426–0.433	0.649^*^	0.646–0.652	0.561^*^	0.558–0.564	0.648^*^	0.644–0.653
5th	0.416^*^	0.412–0.420	0.560^*^	0.556–0.564	0.323^*^	0.320–0.326	0.485^*^	0.482–0.488	0.495^*^	0.492–0.497	0.639^*^	0.635–0.643
**Education level**												
Low	1.000	1.000	1.000	1.000	1.000	1.000	1.000	1.000	1.000	1.000	1.000	1.000
Medium	0.558^*^	0.554–0.561	0.635^*^	0.632–0.664	0.536^*^	0.532–0.539	0.882^*^	0.877–0.896	0.967^*^	0.962–0.972	1.063^*^	1.057–1.069
High	0.353^**^	0.350–0.356	0.417^*^	0.414–0.420	0.445^*^	0.443–0.449	0.580^*^	0.577–0.584	0.674^*^	0.670–0.677	0.740^*^	0.739–0.745
**Health insurance**												
Yes	1.000	1.000	1.000	1.000	1.000	1.000	1.000	1.000	1.000	1.000	1.000	1.000
No	2.284^*^	2.268–2.301	2.182^*^	2.169–2.195	1.635^*^	1.625–1.645	1.572^*^	1.566–1.579	1.871^*^	1.862–1.880	1.933^*^	1.922–1.945
**Physical disability**												
None	1.000	1.000	1.000	1.000	1.000	1.000	1.000	1.000	1.000	1.000	1.000	1.000
Moderate	1.091^*^	1.083–1.098	1.239^*^	1.232–1.246	0.820^*^	0.814–0.826	1.145^*^	1.140–1.151	0.966^*^	0.962–0.970	1.123^*^	1.117–1.129
Severe	2.284^*^	2.267–2.301	3.877^*^	3.796–3.960	2.220^*^	2.174–2.267	3.929^*^	3.847–4.013	1.061^*^	1.045–1.078	0.791^*^	0.777–0.806
**Marital status**												
Single	1.000	1.000	1.000	1.000	1.000	1.000	1.000	1.000	1.000	1.000	1.000	1.000
Not single	0.632^*^	0.629–0.636	0.612^*^	0.610–0.615	0.710^*^	0.707–0.714	0.752^*^	0.750–0.754	0.833^*^	0.830–0.836	0.847^*^	0.844–0.850
**Gender**												
Men									1.000	1.000	1.000	1.000
Women									1.045^*^	1.042–1.049	1.384^*^	1.380–1.389

*The income quintiles were (in Argentinian pesos): 1st quintile (0–8,599); 2nd quintile (8,600–14,999); 3rd quintile (15,000–19,999); 4th quintile (20,000–29,999); 5th quintile (30,000 and above)*.

*Tests for trend across the ordering levels were performed in logistic regression models by assigning the score j to the jth level of the variable selected. All the p-values were P <0.0000 with the exception of the value of sex and use of colorectal cancer screening that was not significant (p = 0.290 for never screened for colorectal cancer and sex)*.

* *p ≤ 0.0001*.

** *p ≤ 0.011*.

*Multiple logistic regressions were used adjusting for age, province of residence, size of city of residence, education level (low, medium or high), self-reported health status (yes or no), physical disability (none, moderate or severe), marital status (single or not single), income (<8,600, 8,600–14,999, 15,000–19,999, 20,000–29,999, ≥30,000) or sex*.

### Measure of Pap Smear Underuse

Eleven thousand six hundred and two women over 35 years old responded the questions on cervical cancer screening. Higher income and education level were inversely related with never having undergone a Pap smear (Table 2) across all income and education level. Similar results were obtained when analyzing data of not having undergone a Pap smear in the previous 2 years. Women with medium and high education levels had higher odds than low educated women to having undergone a Pap smear in the previous 2 years. Severe disability was also strongly associated with never having had a Pap smear and not having had one in the previous 2 years. As observed with mammography, uninsured and single women had higher odds to being underusers of cervical cancer screening (see Table 2).

### Measure of Colorectal Cancer Screening Underuse

Twelve thousand one hundred and twenty two persons (both men and women) responded the questions on colorectal cancer screening. People in quintile 5 and people with high level of education had lower odds to never having undergone colorectal cancer screening. Wealthier people had higher odds to having undergone colorectal cancer screening in the previous 2 years. Education level, disability, health insurance coverage, marital status, and gender had an impact on colorectal cancer screening underuse. However, the general use of colorectal cancer screening was generally low across the sample (see also Table 1).

### Progress in Participation in Cancer Screening: 2013–2018

Table 3 shows the comparison of self-reported underuse of cancer screening across income and education levels between 2013 and 2018. Breast cancer experimented the highest reduction in underuse with the percentage of women that never had had a mammography going down from 23.70 to 18.68%. The highest reduction was observed among women of lower income and among those with medium education level. The percentage of women that had not received a mammography in the last two years went down from 46.87 to 43.33%. This reduction was particularly significant in women of lower income (Q1 and Q2) but it also occurred in women across all income and education levels. The percentage of women that had never undergone a Pap smear was slightly reduced from 13.70 to 11.40%, but there were only small changes in the percentage of women reporting no Pap smear in the last 2 years. Finally, the underuse of colorectal cancer screening went down from 77.74 to 69.34%, with the most significant reduction occurring among people of higher income and those with medium education level. Participation in colorectal cancer screening in the last two years continued to be low in the 2018 survey with an overall 3.80% reduction. Almost all changes in participation across the two surveys were statistically significant (Chi-squared test, *p* ≤ 0.001).

**Table 3:** Variation in breast, cervical, and colorectal cancer screening underuse in Argentina between 2013 and 2018.

**Self-reported participation on breast cancer screening (women over 50)**						
	**Never Mammography** ***n*** **, (%)**			**No mammography in the last 2 years** ***n*** **, (%)**		
	**2013**	**2018**	**Change**	**2013**	**2018**	**Change**
**Overall**	**1,652 (23.70)**	**1,321 (18.68)**	**−5.02**	**3,267 (46.87)**	**3,064 (43.33)**	**−3.54**
**By income (Quintiles)**
1st	424 (38.18)	322 (31.88)	−6.30^*^	665 (59.89)	552 (54.55)	−5.34^**^
2nd	470 (31.03)	399 (24.58)	−6.45^*^	845 (55.81)	837 (51.69)	−4.12^**^
3rd	365 (23.89)	263 (18.25)	−5.64^*^	714 (46.66)	637 (44.14)	−2.52^***^
4th	239 (17.44)	220 (13.71)	−3.73^*^	591 (43.12)	646 (40.27)	−2.85^**^
5th	154 (10.53)	117 (8.40)	−2.13^*^	452 (30.94)	392 (28.16)	−2.78^**^
**By education level**
Low	569 (38.92)	422 (35.82)	−3.10^*^	957 (65.46)	754 (64.00)	−1.46^*^
Medium	751 (26.30)	587 (20.99)	−5.31^*^	1,462 (51.15)	1,356 (48.53)	−2.62^*^
High	332 (12.60)	312 (10.07)	−2.53^*^	848 (32.17)	954 (30.78)	−1.39^*^
**Self–reported participation on cervical cancer screening (women over 35)**						
	**Never PAP smear (%)**			**No PAP smear in the last 2 years (%)**		
	**2013**	**2018**	**Change**	**2013**	**2018**	**Change**
**Overall**	**1,558 (13.70)**	**1,323 (11.40)**	**−2.30**	**4,589 (40.35)**	**4,587 (39.53)**	**−0.82**
**By income (Quintiles)**
1st	434 (19.08)	369 (18.11)	−0.97^*^	1,068 (46.94)	971 (47.56)	0.62^*^
2nd	421 (18.04)	366 (14.85)	−3.19^*^	1,112 (47.63)	1,157 (46.93)	−0.70^*^
3rd	305 (13.59)	242 (10.54)	−3.05^*^	943 (41.98)	923 (40.19)	−1.79^*^
4th	232 (10.74)	205 (8.22)	−2.52^*^	831 (38.45)	910 (36.50)	−1.95^*^
5th	166 (7.02)	141 (6.09)	−0.93^*^	635 (26.84)	630 (27.21)	0.37^*^
**By education level**
Low	445 (26.52)	344 (24.68)	−1.84^*^	1,075 (64.09)	859 (61.62)	−2.47^*^
Medium	658 (15.36)	531 (13.06)	−2.30^*^	2,033 (47.44)	1,934 (47.59)	0.15^*^
High	455 (8.44)	448 (7.29)	−1.15^*^	1,485 (27.52)	1,794 (29.19)	1.67^*^
**Self–reported participation on colorectal cancer screening (men/women over 50)**						
	**Never colorectal cancer screening (%)**			**No screening in the last 2 years (%)**		
	**2013**	**2018**	**Change**	**2013**	**2018**	**Change**
**Overall**	**9,316 (77.74)**	**8,405 (69.34)**	**−8.40**	**10,520 (86.75)**	**10,055 (82.95)**	**−3.80**
**By income (Quintiles)**
1st	1,629 (83.90)	1,359 (79.75)	−4.15^*^	1,761 (90.72)	1,529 (89.28)	−1.44^*^
2nd	2,022 (80.83)	1,967 (74.90)	−5.93^*^	2,237 (89.42)	2,294 (86.99)	−2.43^*^
3rd	2,039 (77.41)	1,749 (71.02)	−6.39^*^	2,302 (87.45)	2,069 (83.68)	−3.77^*^
4th	1,722 (74.10)	1,789 (66.43)	−7.67^*^	1,984 (85.39)	2,177 (80.51)	−4.88^*^
5th	1,904 (69.82)	1,541 (59.74)	−10.08^*^	2,236 (81.99)	1,986 (76.76)	−5.23^*^
**By education level**
Low	2,041 (81.87)	1,501 (75.20)	−6.67^*^	2,237 (89.75)	1,731 (86.70)	−2.96^*^
Medium	4,069 (79.74)	3,615 (72.25)	−7.49^*^	4,528 88.74	4,258 (85.11)	−3.63^*^
High	3,206 (70.72)	3,289 (64.91)	−5.81^*^	3,755 (82.82)	4,025 (79.43)	−3.39^*^

*The income quintiles were (in Argentinian pesos): 1st quintile (0–8,599); 2nd quintile (8,600–14,999); 3rd quintile (15,000-19,999); 4th quintile (20,000–29,999); 5th quintile (30,000 and above)*.

* *p ≤ 0.0001*.

** *p ≤ 0.001*.

*** *p ≤ 0.002*.

**Figure 1:**
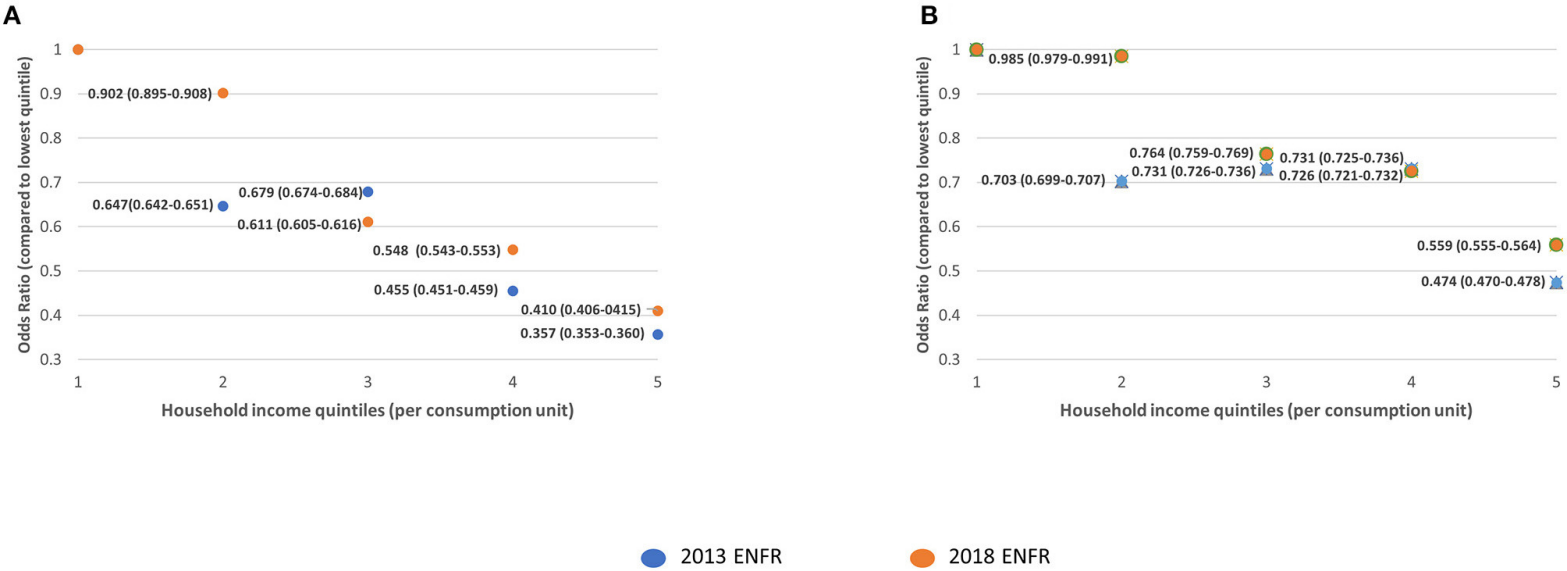
Comparison of the adjusted odds ratios for participation in breast cancer screening across income quintiles. **(A)** Never had a mammography. **(B)** Did not have mammography in the previous 2 years.

**Figure 2:**
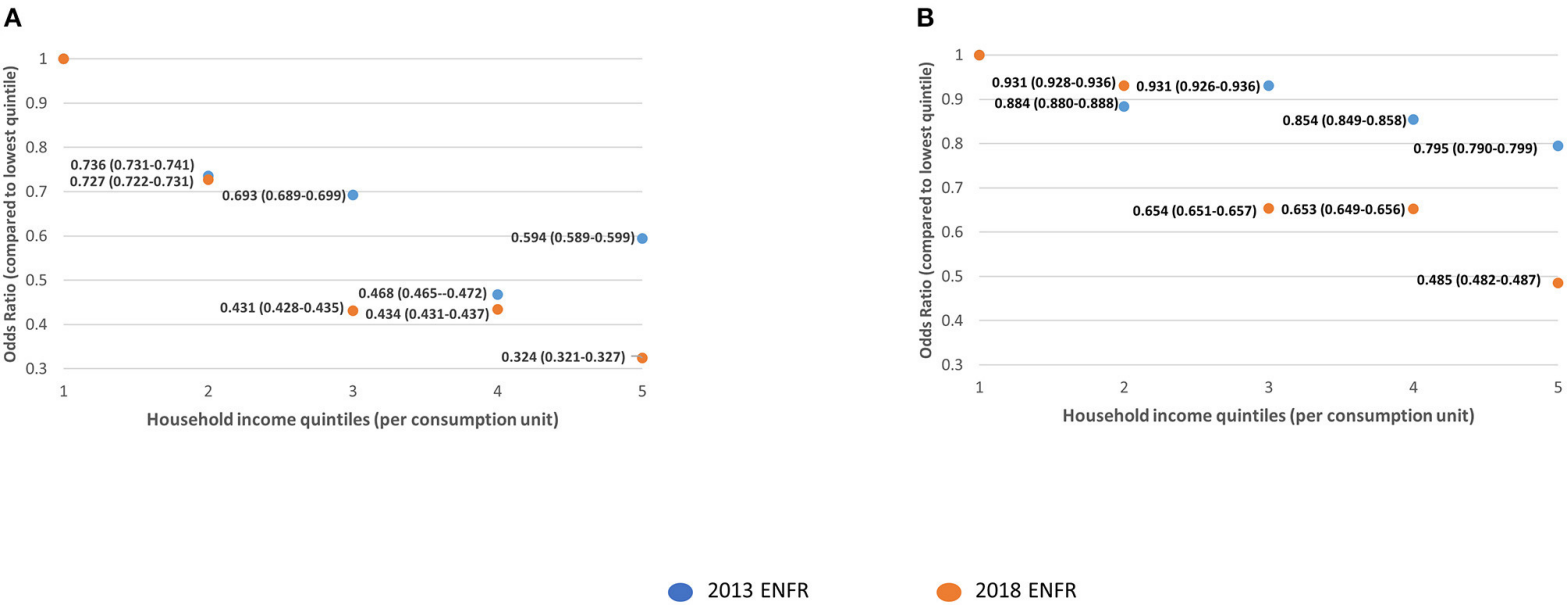
Comparison of the adjusted odds ratios for participation in cervical cancer screening across income quintiles. **(A)** Never had a Pap Smear. **(B)** Did not have a Pap Smear in the previous 2 years.

**Figure 3:**
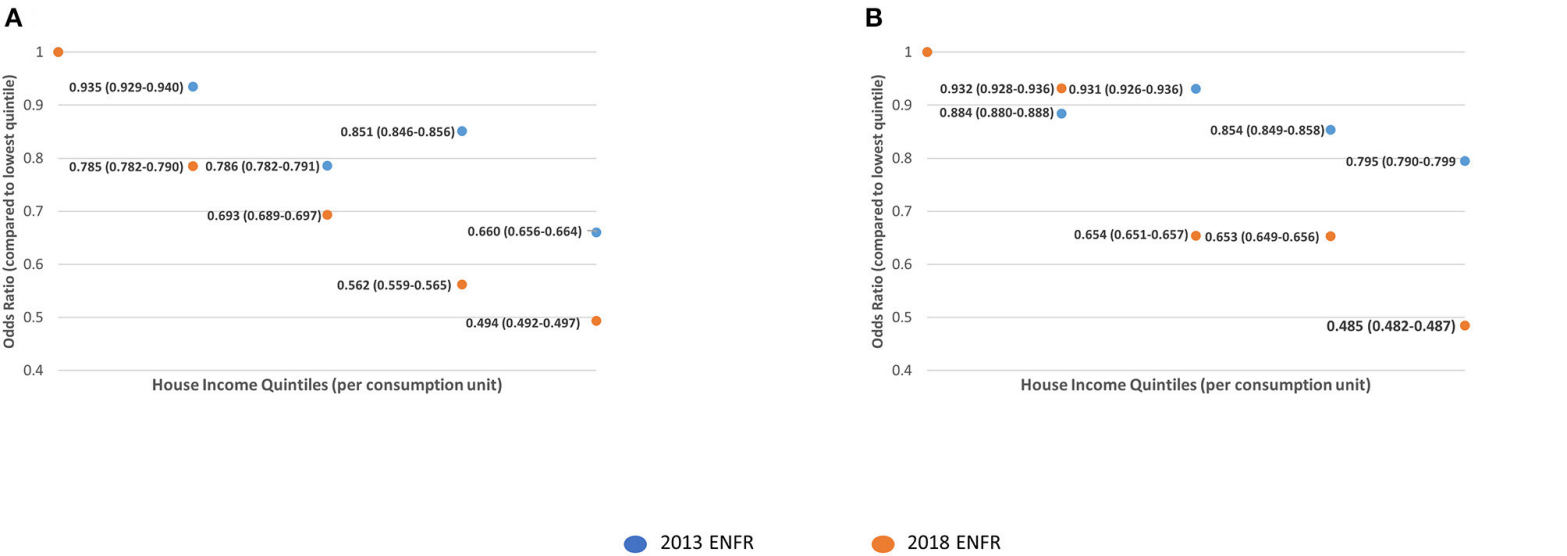
Comparison of the adjusted odds ratios for participation in colorectal cancer screening across income quintiles. **(A)** Never had a colorectal cancer screening. **(B)** Did not have colorectal cancer screening in the previous 2 years.

Figures 1–**3** show the comparisons of the adjusted odds ratios across income quintiles of the two measures of breast, cervical, and colorectal cancer screening underuse between 2013 and 2018.

The participation in breast cancer screening in women older than 50 was generally more equal across income quintiles in 2018, as compared to 2013 (Figure 1A). This flattening in the differences in participation across quintiles seems to be driven by the higher decrease in screening underuse in quintiles 1 and 2. Regarding cervical cancer screening, the differences in the participation between 2013 and 2018 were reduced between the first two quintiles. However, these differences were widened when comparing quintiles 3, 4, and 5 with quintile 1 (Figure 2). This probably reflects a more intense decrease in underuse for women in the higher income quintiles. Regarding colorectal cancer screening, the most significant reductions in odds of underuse were observed across the top quintiles, leading to a widening of disparities in screening uptake between 2013 and 2018 (Figure 3).

## Discussion

We found income, education, health insurance, marital status, and physical disability to be associated with two measures of underuse (i.e., never use or infrequent use) of breast, cervical, and colorectal cancer screening in Argentina. This is consistent with recent evidence demonstrating widening socioeconomic inequalities across Latin America (34). The findings indicate that in the last few years there has been progress toward reducing underuse of participation in screening, but this progress is not equal across all income and education levels. By identifying populations who are consistently not being adequately reached by cancer screening services, including uninsured women and women with physical disability, the findings make a unique contribution to the body of evidence on the socioeconomic determinants of cancer screening and can be used to inform strategies to bridge disparities in cancer screening uptake in Argentina.

The analysis of the ENFR database is cross-sectional and therefore causation between the independent and dependent variables could not be established. Furthermore, the ENFR relies on information provided through an interviewer-administered questionnaire, which leaves the instrument open to interviewer bias; however, there is no relevant information on this aspect. It is also important to note that because the ENFR only collects data from villages/towns with more than 5,000 inhabitants, the information from rural communities with poorer access to health care services was not analyzed.

These limitations were partly addressed by following a rigorous process in which the independent variables were selected based on previous reported evidence in Latin America (7) and then analyzed using data from a reliable dataset, representative of the urban Argentinean population. According to the most recent estimations published by the World Bank (https://data.worldbank.org/indicator/SP.URB.TOTL.IN.ZS?locations=AR), 91,9% of the Argentinean population lives in urban areas of over 2,000 inhabitants (35).

Compared to the results from a previous study that used data from the 2005 and 2009 ENFR (17), the self-reported participation in mammography screening has increased across all socioeconomic levels. This could be a result of the initiation of the national breast cancer programme in 2011 (22), which introduced a systematic strategy on breast cancer prevention.

The results also show that disparities between women with different income levels in accessing cervical cancer screening had reduced in 2013 compared to a previous study (17). However, when analyzing data from 2018 these differences with respect to quintile 1 widened again probably driven by a more intense growth in participation by women in the higher quintiles. This may be an indicator of increased utilization of cervical cancer screening in the country by a part of the population, as a result of the relaunching of the cervical cancer national strategy in 2008 (21).

The percentage of population that had never been screened for colorectal cancer was very high. This could be attributed to the relatively recent introduction in 2013 of the national programme for colorectal cancer screening. Reductions in poor uptake of colorectal cancer screening between 2013 and 2018 were mostly observed among people with high income, indicating that availability of services alone is not enough for the equitable use of screening services across the population.

Similarly to other countries in Latin America (7), being covered by an insurance plan was associated with higher utilization of cancer screening. The Argentinian health system is fragmented, with 16% of the population being covered by private health insurance, 63% by social security, and 36% by the public health care system (9). Access to healthcare services is determined by the type of insurance, and therefore, access to cancer screening may vary across the population (14). Previous evidence suggests that users' perceptions of insurance coverage are not always accurate, leading to cost-related barriers to participation in screening (36).

Low socioeconomic status can lead to structural disadvantage (1); the interactions between low education, low income, disability, and lack of insurance can lead to a series of barriers to accessing health services, including cancer screening. Cancer screening uptake is influenced by a complex nexus of factors (16), and its associations with socioeconomic status are multifaceted, extending from inability to pay for transportation costs or screening tests, or take time off work to attend screening, to poor knowledge regarding cancer screening and reduced referrals by health professionals (1, 37). Solutions developed in and for high-income countries are not necessarily effective elsewhere (38). Understanding context-specific barriers to screening uptake is an essential element of cancer screening programmes (39).

## Conclusions

Our data show that Argentina has progressed toward increasing cancer screening uptake since 2013 However, there is an improvement margin to ensure that the increase is more equitable. To make further progress toward reducing avoidable cancer deaths, the country should develop information campaigns that proactively reach those populations that are not benefiting from regular cancer screening. This, however, is not enough; cancer screening services need to be available, accessible, and affordable, and disability-inclusive in order for people to be able to participate (40). This will need action at the local level, since despite the existence of national strategies cancer screening, the Ministry of Health provides recommendations and the provinces are responsible of their implementation. Evidence suggests that community health workers can play a role in cancer screening (41, 42). Further increase in cancer screening coverage can be achieved by developing population-based screening programs to recruit populations from different age groups and socioeconomic levels through, for example, linking cancer screening to services that are used by these populations and upscaling the introduction of approaches such as HPV self-sampling, mobile mammography, and colonoscopy services. Existing barriers to access need to be addressed, so that screening services are equitably used and they meet their ultimate goal of reducing cancer morbidity and mortality.

## Data Availability Statement

Publicly available datasets were analyzed in this study. The datasets analyzed in the current study are available from the National Institute of Statistics and Censuses of Argentina https://www.indec.gob.ar/indec/web/Institucional-Indec-BasesDeDatos-2.

## Ethics Statement

We performed a secondary analysis of cross-sectional data from the 2018 National Survey of Risk Factors of Argentina (known as ENFR in Spanish) (28), and we also compared this with data from the 2013 ENFR. All microdata obtained from the ENFR were freely available in the public domain (29). The Research Governance & Integrity Office of the London School of Hygiene and Tropical Medicine assessed that this project did not require ethical approval.

## Author Contributions

BN-B conceived the final research question and aims and objectives, reviewed the literature, and carried out the analysis. BN-B and DS jointly designed the study, devised the analysis strategy, and drafted the manuscript. Both authors read and approved the final manuscript.

## Conflict of Interest

The authors declare that the research was conducted in the absence of any commercial or financial relationships that could be construed as a potential conflict of interest.

## Publisher's Note

All claims expressed in this article are solely those of the authors and do not necessarily represent those of their affiliated organizations, or those of the publisher, the editors and the reviewers. Any product that may be evaluated in this article, or claim that may be made by its manufacturer, is not guaranteed or endorsed by the publisher.

## Abbreviations

ENFR: Encuesta Nacional de Factores de Riesgo (National Survey of Risk Factors of Argentina)
HPV: Human Papillomavirus.
