# Supplementary material for: Socioeconomic Determinants of Participation in Cancer Screening in Argentina: A Cross-Sectional Study
Source: Front Public Health. 2021 Aug 24;9:699108. doi: 10.3389/fpubh.2021.699108 (PMC8423085; doi:10.3389/fpubh.2021.699108)
Supplement: Supplementary file 2 [file Table_2.DOCX]

**SUPPLEMENTARY MATERIAL**

**Supplementary material 1. Socioeconomic characteristics of the sample population (2018)**

|  | Women older than 35  (n=11,602) | Women older than 50  (n=7,070) | Men/women older than 50 (n=12,122) |
| --- | --- | --- | --- |
| **Income (Quintiles)** |  |  |  |
| 1^st^ | 2,037 | 1,010 | 1,714 |
| 2^nd^ | 2,463 | 1,623 | 2,637 |
| 3^rd^ | 2,294 | 1,441 | 2,474 |
| 4^th^ | 2,493 | 1,604 | 2,705 |
| 5^th^ | 2,315 | 1,392 | 2,592 |
| **Education level** |  |  |  |
| Low | 1,394 | 1,178 | 2009 |
| Medium | 4,066 | 2,796 | 5031 |
| High | 6,142 | 3,096 | 5,082 |
| **Health insurance** |  |  |  |
| Yes | 9,196 | 6,130 | 10,181 |
| No | 2,406 | 940 | 1,941 |
| **Marital status** |  |  |  |
| Other | 5,441 | 2,729 | 5,795 |
| Single | 6,161 | 4,341 | 6,325 |
| **Self-reported health status** | |  |  |
| Excellent | 794 | 375 | 687 |
| Very good | 2,527 | 1,261 | 2,248 |
| Good | 4,827 | 2,923 | 5,157 |
| Neither good or bad | 2,951 | 2,100 | 3,416 |
| Bad | 503 | 411 | 614 |
| **Physical disability** |  |  |  |
| None | 9,298 | 5,070 | 9,106 |
| Moderate | 2,236 | 1,910 | 2,868 |
| Severe | 98 | 90 | 148 |
| **Gender** | | | |
| Male | n/a | n/a | 5052 |
| Female | n/a | n/a | 7,070 |
| **Province of residence** | | | |
| City of Buenos Aires | 586 | 385 | 656 |
| Buenos Aires | 2,347 | 1,480 | 2,579 |
| Catamarca | 323 | 187 | 335 |
| Córdoba | 764 | 497 | 841 |
| Corrientes | 414 | 244 | 416 |
| Chaco | 387 | 221 | 378 |
| Chubut | 388 | 233 | 377 |
| Entre Ríos | 676 | 424 | 692 |
| Formosa | 399 | 242 | 416 |
| Jujuy | 381 | 196 | 344 |
| La Pampa | 262 | 174 | 279 |
| La Rioja | 288 | 159 | 264 |
| Mendoza | 422 | 280 | 459 |
| Misiones | 409 | 233 | 410 |
| Neuquén | 290 | 169 | 299 |
| Río Negro | 568 | 348 | 598 |
| Salta | 449 | 263 | 464 |
| San Juan | 270 | 167 | 277 |
| San Luis | 392 | 255 | 430 |
| Santa Cruz | 199 | 99 | 189 |
| Santa Fe | 679 | 413 | 740 |
| Santiago del Estero | 236 | 117 | 206 |
| Tucumán | 364 | 226 | 373 |
| Tierra del Fuego | 109 | 58 | 100 |

**Supplementary material 2. Socioeconomic characteristics of the sample population (2013)**

|  | Women older than 35  (n=11,816) | Women older than 50  (n=6,972) | Men/women older than 50 (n=11,983) |
| --- | --- | --- | --- |
| **Income (Quintiles)** |  |  |  |
| 1^st^ | 2,353 | 1,109 | 1,907 |
| 2^nd^ | 2,391 | 1,516 | 2,467 |
| 3^rd^ | 2,358 | 1,510 | 2,598 |
| 4^th^ | 2,250 | 1,367 | 2,286 |
| 5^th^ | 2,436 | 1,453 | 2,687 |
| **Education level** |  |  |  |
| Low | 1,754 | 1,462 | 2,449 |
| Medium | 4,421 | 2,855 | 5,080 |
| High | 5,641 | 2,655 | 4,454 |
| **Health insurance** |  |  |  |
| Yes | 9,343 | 5,986 | 9,998 |
| No | 2,473 | 986 | 1,985 |
| **Marital status** |  |  |  |
| Other | 5,876 | 2,883 | 6,141 |
| Single | 5,940 | 4,089 | 5,842 |
| **Self-reported health status** | |  |  |
| Excellent | 826 | 339 | 642 |
| Very good | 2,091 | 867 | 1,602 |
| Good | 5,043 | 2,907 | 5,219 |
| Neither good or bad | 3,318 | 2,417 | 3,830 |
| Bad | 538 | 442 | 690 |
| **Physical disability** |  |  |  |
| None | 9,567 | 5,045 | 9,105 |
| Moderate | 2,156 | 1,844 | 2,761 |
| Severe | 93 | 83 | 117 |
| **Gender** | | | |
| Male | n/a | n/a | 5,011 |
| Female | n/a | n/a | 6,972 |
| **Province of residence** | | | |
| City of Buenos Aires | 307 | 209 | 354 |
| Buenos Aires | 2,030 | 1,269 | 2,116 |
| Catamarca | 300 | 161 | 290 |
| Córdoba | 723 | 458 | 790 |
| Corrientes | 428 | 264 | 417 |
| Chaco | 333 | 194 | 318 |
| Chubut | 482 | 252 | 472 |
| Entre Ríos | 549 | 358 | 569 |
| Formosa | 355 | 152 | 317 |
| Jujuy | 363 | 200 | 353 |
| La Pampa | 363 | 213 | 372 |
| La Rioja | 350 | 188 | 326 |
| Mendoza | 539 | 351 | 572 |
| Misiones | 322 | 163 | 298 |
| Neuquén | 344 | 200 | 339 |
| Río Negro | 643 | 393 | 676 |
| Salta | 428 | 217 | 383 |
| San Juan | 357 | 232 | 400 |
| San Luis | 364 | 210 | 364 |
| Santa Cruz | 323 | 170 | 306 |
| Santa Fe | 780 | 504 | 856 |
| Santiago del Estero | 342 | 175 | 314 |
| Tucumán | 469 | 275 | 480 |
| Tierra del Fuego | 322 | 164 | 301 |

**Supplementary material 3. Age distribution of the sample population in the 2013 and 2018 ENFR**

|  | **2013** | | | **2018** | | |
| --- | --- | --- | --- | --- | --- | --- |
| **Age Range** | **Women** | **Men** | **Total** | **Women** | **Men** | **Total** |
| **35-40** | 1,932 | N/A | N/A | 1,735 | N/A | N/A |
| **40-45** | 1,586 | N/A | N/A | 1,474 | N/A | N/A |
| **45-50** | 1,326 | N/A | N/A | 1,323 | N/A | N/A |
| **50-55** | 1,309 | 1,110 | 2,419 | 1,267 | 984 | 2,251 |
| **55-60** | 1,119 | 963 | 2,082 | 1,186 | 910 | 2,096 |
| **60-65** | 1,199 | 907 | 2,106 | 1,146 | 920 | 2,066 |
| **65-70** | 1,022 | 721 | 1,743 | 1,119 | 799 | 1,918 |
| **70-75** | 840 | 546 | 1,386 | 883 | 602 | 1,485 |
| **75-80** | 670 | 374 | 1,044 | 677 | 448 | 1,125 |
| **80-85** | 506 | 245 | 751 | 470 | 229 | 699 |
| **85-90** | 231 | 115 | 346 | 229 | 116 | 345 |
| **Older than 90** | 76 | 30 | 106 | 93 | 44 | 137 |
